# Method of Changing Running Direction of Cheetah-Inspired Quadruped Robot

**DOI:** 10.3390/s22249601

**Published:** 2022-12-07

**Authors:** Meng Ning, Jun Yang, Ziqiang Zhang, Jun Li, Zhi Wang, Longxing Wei, Pengjin Feng

**Affiliations:** 1 Jiangsu Key Laboratory of Advanced Food Manufacturing Equipment & Technology, School of Mechanical Engineering, Jiangnan University, Wuxi 214122, China; 2 Faculty of Materials and Manufacturing, Beijing University of Technology, Beijing 100124, China

**Keywords:** quadruped robot, change of running direction, dynamic model, stability index system, simulation analysis

## Abstract

The rapid change of motion direction during running is beneficial to improving the movement flexibility of the quadruped robot, which is of great relevance to its research. How to make the robot change its motion direction during running and achieve good dynamic stability is a problem to be solved. In this paper, a method to change the running direction of the cheetah-inspired quadruped robot is proposed. Based on the analysis of the running of the cheetah, a dynamic model of the quadruped robot is established, and a two-level stability index system, including a minimum index system and a range index system, is proposed. On this basis, the objective function based on the stability index system and optimization variables, including leg landing points, trunk movement trajectory, and posture change rule, are determined. Through these constraints, the direction changes with good dynamic stability of the cheetah-inspired quadruped robot during running is realized by controlling the leg parameters. The robot will not roll over during high-speed movement. Finally, the correctness of the proposed method is proven by simulation. This paper provides a theoretical basis for the quadruped robot’s rapid change of direction in running.

## 1. Introduction

Most quadrupeds have the ability to run fast. For example, the cheetah is the fastest-running land animal in the world, and its speed can reach 104.4 km/h [1]. Antilocapra americana can run very fast, up to 100 km/h, and has good endurance [2]. In particular, to catch a fast-moving target or escape quickly, the running direction of the creature is not constant, thus its running is no longer a plane motion but a motion in a 3D space. Therefore, for the quadruped robot, how to achieve a rapid change of motion direction in running is a problem to be solved [3].

Many researchers have studied the movement mechanism of the quadruped during running [4]. For example, Kamimura et al. [5] hypothesized that the three characteristics of the small vertical movement of their center of mass, small whole-body pitching movement, and large spine bending movement enhance the running ability of the cheetah. The hypothesis was then verified by a model with a spine joint and a torsional spring. In addition, the running of bipedal creatures, such as birds [6,7] or humans [8,9], has also been studied. On this basis, many researchers have studied the running of bio-inspired quadruped robots. To make the quadruped robot have good dynamic performance in running, the research mainly focuses on structural design [10,11], a control algorithm based on the dynamic model [12,13,14], an energy transfer mechanism [15], and environmental adaptability [16,17,18]. In terms of prototype, the most representative quadruped robot with running ability is the Cheetah robot developed by the Massachusetts Institute of Technology (MIT). Based on the research on the design principles for highly efficient legged robots and hierarchical controllers, the running speed of the Cheetah robot can reach 6 m/s and has good dynamic performance [19,20,21,22]. In addition to quadruped robots, many researchers have studied the running of biped robots [23,24] and hexapod robots [25,26], and have achieved good research results.

Creatures often do not run in only one direction, and they have the ability to move at high speed in 3D space [27,28]. The research on the motion abilities of bio-inspired robots has also expanded from plane motion to 3D space [29,30]. For examples, Di Carlo et al. [22] presented the implementation of model predictive control (MPC) to determine ground reaction forces for a torque-controlled quadruped robot, and the developed MIT Cheetah 3 can realize a full 3D gallop. Sullivan et al. [31] studied the effects of varying step width on the 3D running stability of a bipedal amputee-inspired robot. The research results showed that to obtain narrower step widths, as seen in human locomotion, a roll and yaw control would be needed. In addition to controlling the motion parameters of the robot itself, the robot can change motion direction during running by using auxiliary mechanisms. For example, Kim et al. [32] were inspired by a basilisk lizard’s ability to run and steer on water surfaces for a hexapedal robot, which can steer on water by rotating its tail, and the controlled steering locomotion was stable. Kohut et al. [33] presented a running robot that used aerodynamic forces to turn. The research results showed that the robot is capable of stably turning in a 1.2 m radius at 1.6 ms, and the aerodynamic steering is superior for high-speed turns at high forward velocity. In particular, jumping is also a high-speed movement. Many researchers have studied the structure [34,35,36] and control algorithms [37,38] of the robot so that it can achieve fast steering when jumping.

For quadruped robots with running ability, the existing research mainly focuses on running in a plane. Research on the high-speed motion mechanism of the quadruped robot in 3D space is relatively rare. The difficulty of research on the 3D running of robots is mainly reflected in two aspects: the motion of the robot in 3D space involves many dynamic performance indices and variables to be optimized, and the coupling degree between them is high [39]; conversely, the change of direction in the high-speed motion of the robot can easily cause sudden changes in performance indices [40]. Guaranteeing the stable high-speed movement of the robot is difficult. To make the robot achieve good dynamic stability in high-speed steering, taking the steering running of the cheetah as a reference, a method of changing the running direction of a bio-inspired robot is proposed in this paper. A two-level stability index system, including minimum index system and range index system, is established based on the dynamic model of the robot, and the optimization variables, including leg landing points, trunk movement trajectory, and posture change rule, are determined. Then, the optimal leg input parameters can be obtained based on the improved bee colony algorithm. The analysis results show that the robot can turn quickly while running and has good dynamic stability by using the proposed method.

The remainder of the paper is structured as follows. Section 2 establishes the dynamic model and stability index system, and presents the optimization method of leg parameters. Section 3 shows examples to illustrate the feasibility of the method. Finally, Section 4 discusses the results. This paper provides a theoretical basis for the realization of rapid steering in the running of the quadruped robot.

## 2. Methods

### 2.1. Research Objectives

**Figure 1:**
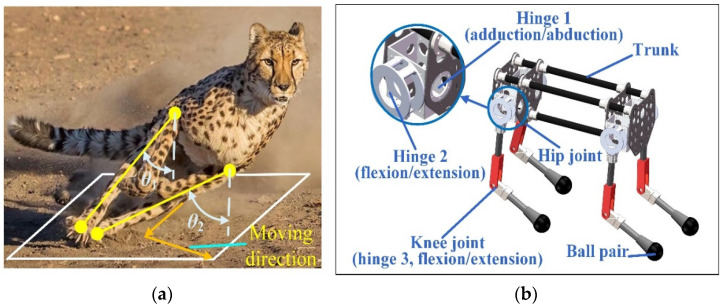
(**a**) Moment when the cheetah changes its movement direction during running. The legs of the cheetah have larger adduction/abduction angles than the cheetah moving in one plane. (**b**) 3D model of the cheetah-inspired quadruped robot. The hip joint has 2 DOFs, and the knee joint has 1 DOF.

Cheetahs often need to change movement direction frequently during hunting. When the cheetah runs in a plane, the angle of leg adduction/abduction is almost zero. When the cheetah needs to change the motion direction during high-speed movement, the adduction/abduction angle is large. Figure 1a shows *θ*_1_ and *θ*_2_ are the angles between the leg and the vertical direction in the front view, which are 36.6° and 64.2°, respectively, in the illustrated state. With the cooperation of muscle-driving forces, the cheetah can realize steering movement during running. In particular, for the movement gait, cheetahs use a rotatory gallop with the footfall order of right fore, left fore, left hind, and right hind during curve running [41].

The 3D model of the cheetah-inspired quadruped robot is shown in Figure 1b. The hip joints of each leg have 2 degrees of freedoms (DOF) for adduction/abduction and flexion/extension movements. The axes of the two hinges intersect. The knee joint has 1 DOF for flexion/extension movement. In addition, the leg is in point contact with the ground, which can be equivalent to a 3-DOF ball pair. At this time, each leg has 6 DOFs and no constraints on the trunk. By controlling the leg postures and driving forces of the cheetah-inspired robot, the robot can simulate the cheetah to change motion direction quickly during high-speed motion.

### 2.2. Establishment of Dynamic Model

**Figure 2:**
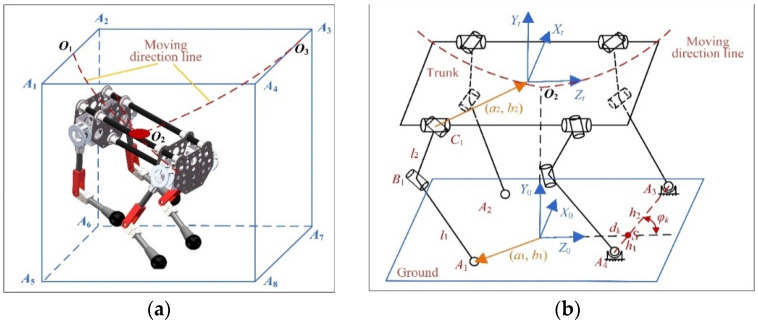
(**a**) Trajectory of the trunk of the cheetah-inspired quadruped robot in the process of changing the motion direction in the leg landing phase. The trajectory *O*_1_*O*_2_ of the center of mass of the trunk in the descending phase and the trajectory *O*_2_*O*_3_ in the ascending phase are not coplanar. (**b**) Mechanism diagram of the cheetah-inspired quadruped robot.

During running, the cheetah’s two forelegs land first and its two hindlegs land later [41]. For simplicity, the two forelegs are assumed to land simultaneously. When the trunk moves to the lowest point, both forelegs leave the ground at the same time, and both hindlegs land. The trajectory of the trunk during the turning of the robot in the leg landing phase is shown in Figure 2a. When the forelegs of the robot land on the ground, its trunk moves along the trajectory *O*_1_*O*_2_ (*O*_1_ is the position of the center of mass of the trunk at the moment when the forelegs land, and *O*_2_ is the lowest point of the trunk). When the center of mass of the trunk reaches *O*_2_, the movement direction of the trunk is changed, and the robot leaves the ground along the trajectory *O*_2_*O*_3_ (*O*_3_ is the position of center of mass of the trunk at the moment when the hindlegs leave the ground). In the following text, the “trunk descending phase” and “trunk ascending phase” refer to the above two processes. Trajectories *O*_1_*O*_2_ and *O*_2_*O*_3_ are not coplanar, and the trajectory is not necessarily a straight line. The mechanism diagram of the cheetah-inspired quadruped robot is shown in Figure 2b. The coordinate origin of the fixed coordinate system *O*_0_–*X*_0_*Y*_0_*Z*_0_ coincides with the projection point of the lowest point *O*_2_ of the trunk motion trajectory on the ground. The directions of the coordinate axes are shown in Figure 2b. The coordinate origin of the moving coordinate system *O_t_–X_t_Y_t_Z_t_* coincides with the geometric center of the trunk, and the friction between the legs and the ground during the movement is ignored.

First, the motion law of the trunk must be determined. Trajectories *O*_1_*O*_2_ and *O*_2_*O*_3_ can be expressed as

(1) $$S_{t}\left(x,y,z\right)=S_{t}\left(\sum_{q=0}^{n}d_{qi}t^{q},\sum_{q=0}^{n}e_{qi}t^{q},\sum_{q=0}^{n}f_{qi}t^{q}\right),\;i=1,\;2\;$$

where *t* is the time, *a_q_*, *b_q_*, and *c_q_* are the polynomial coefficients, and (*x*_1_, *y*_1_, *z*_1_) and (*x*_2_, *y*_2_, *z*_2_) are the coordinates of points *O*_1_ and *O*_2_, respectively. Equation (1) can also reflect the change of velocity and acceleration of the trunk by derivation. In particular, some boundary conditions are known. For example, at the moment when the forelegs of the robot land on the ground and the trunk reaches the lowest point *O*_2_, the position and the velocity of the center of mass of the trunk are known. The velocity of the trunk at point *O*_1_ is also known according to the motion parameters of the previous cycle (a running cycle is defined from the moment of robot landing to the next landing moment). Therefore, a coupling relationship between the polynomial coefficients may exist.

During the movement of the trunk along trajectories *O*_1_*O*_2_ and *O*_2_*O*_3_, the posture of the trunk can be represented by the *ZYX* Euler angle.

(2) $$Q=\left(\begin{array}{cccc} c\alpha c\beta & c\alpha s\beta s\gamma -s\alpha c\gamma & c\alpha s\beta c\gamma +s\alpha s\gamma & x\\ s\alpha c\beta & s\alpha s\beta s\gamma +c\alpha c\gamma & s\alpha s\beta c\gamma -c\alpha s\gamma & y\\ -s\beta & c\beta s\gamma & c\beta c\gamma & z\\ 0 & 0 & 0 & 1 \end{array}\right),$$

where “*s*” and “*c*” refer to “sin” and “cos”, respectively, and *α*, *β*, and *γ* are the Euler angles of the motion coordinate system *O_t_*–*X_t_Y_t_Z_t_* relative to the fixed coordinate system *O*_0_–*X*_0_*Y*_0_*Z*_0_. The change of trunk posture can be expressed as a polynomial function:

(3) $$\Phi_{t}\left(\alpha ,\beta ,\gamma \right)=\Phi_{t}\left(\sum_{q=0}^{n}a_{qi}t^{q},\sum_{q=0}^{n}b_{qi}t^{q},\sum_{q=0}^{n}c_{qi}t^{q}\right)\;i=1,\;2$$

Similarly, boundary conditions can be set according to actual requirements to reduce the number of polynomial coefficients to be optimized.

If the position and the posture of the trunk are determined, the leg posture can be reflected through the contact points between the legs and the ground. Taking the foreleg landing as an example, the contact points can be determined by four parameters, *h*_1_, *h*_2_, *d_k_*, and *φ_k_*, as shown in Figure 2b. *h*_1_ and *h*_2_ are the distances from the two landing points to the intersection *S* of *A*_3_*A*_4_ (*A*_3_ and *A*_4_ are the two landing points of forelegs) and the *Z*_0_ axis, respectively. *d_k_* is the distance from the coordinate origin of *O*_0_–*X*_0_*Y*_0_*Z*_0_ to *S*. *φ_k_* is the angle between *A*_3_*A*_4_ and the positive direction of the *Z*_0_ axis. At this time, the coordinates of the two landing points *A*_3_ and *A*_4_ can be expressed as

(4) $$A_{i}=\left[\begin{array}{c} {\left(-1\right)}^{i}h_{i}\sin\varphi_{k}\;0\;d_{k}+{\left(-1\right)}^{i}h_{i}\cos\varphi_{k} \end{array}\right]\;i=3,\;4$$

The calculation method of the landing points is the same for the case of hindleg landing. The kinematic equation of the *i*-th leg can be expressed as

(5) $${\;}^{0}T{}_{t}={\;}^{0}T{}_{i1}{\;}^{i1}T{}_{i2}{\;}^{i2}T{}_{i3}{\;}^{i3}T{}_{ti},$$

where

$${\;}^{0}T_{i1}=\left(\begin{array}{cccc} c\theta_{i1}c\theta_{i2} & c\theta_{i1}s\theta_{i2}s\theta_{i3}-s\theta_{i1}c\theta_{i3} & c\theta_{i1}s\theta_{i2}c\theta_{i3}+s\theta_{i1}s\theta_{i3} & l_{i1}+l_{i3}c\theta_{i1}c\theta_{i2}\\ s\theta_{i1}c\theta_{i2} & s\theta_{i1}s\theta_{i2}s\theta_{i3}+c\theta_{i1}c\theta_{i3} & s\theta_{i1}s\theta_{i2}c\theta_{i3}-c\theta_{i1}s\theta_{i3} & l_{i3}s\theta_{i1}c\theta_{i2}\\ -s\theta_{i2} & c\theta_{i2}s\theta_{i3} & c\theta_{i2}c\theta_{i3} & l_{i2}-l_{i3}s\theta_{i2}\\ 0 & 0 & 0 & 1 \end{array}\right),$$

$${\;}^{i1}T_{i2}=\left(\begin{array}{cccc} s\theta_{i4} & -s\theta_{i4} & 0 & l_{i4}c\theta_{i4}\\ s\theta_{i4} & c\theta_{i4} & 0 & l_{i4}s\theta_{i4}\\ 0 & 0 & 1 & 0\\ 0 & 0 & 0 & 1 \end{array}\right),\;{\;}^{i2}T_{i3}=\left(\begin{array}{cccc} c\theta_{i5} & 0 & s\theta_{i5} & 0\\ s\theta_{i5} & 0 & -c\theta_{i5} & 0\\ 0 & 1 & 0 & 0\\ 0 & 0 & 0 & 1 \end{array}\right),\;{\;}^{i3}T_{ti}=\left(\begin{array}{cccc} c\theta_{i6} & -s\theta_{i6} & 0 & l_{i5}c\theta_{i6}\\ s\theta_{i6} & c\theta_{i6} & 0 & l_{i5}s\theta_{i6}\\ 0 & 0 & 1 & l_{i6}\\ 0 & 0 & 0 & 1 \end{array}\right)$$

*θ_i_*_1_, *θ_i_*_2_, and *θ_i_*_3_ are the rotation angles of the ball pair. *θ_i_*_4_ is the rotation angle of the knee joint. *θ_i_*_5_ and *θ_i_*_6_ are the rotation angles of hinges 2 and 1, respectively, as shown in Figure 1b. (*a_i_*_1_, *b_i_*_1_) is the position vector of point *A_i_* in the fixed coordinate system *O*_0_–*X*_0_*Y*_0_*Z*_0_, *l_i_*_1_ is the length of link *A_i_B_i_*, *l_i_*_2_ is the length of link *B_i_C_i_*, and (*a_i_*_2_, *b_i2_*) is the position vector of point *C_i_* in the moving coordinate system *O_t_*–*X_t_Y_t_Z_t_*. For Equation (5), when the position and the posture of the trunk and the position of the landing points are determined, the joint angle *θ_ij_* (*j* = 1, 2, …, 6) can be obtained by numerical solution. At this time, the position vector of any point on the link can be expressed as

(6) $$r_{ip}=r_{ipx}\vec{i}+r_{ipy}\vec{j}+r_{ipz}\vec{k}$$

On the basis of solving the kinematics, the dynamic model of the robot should be established. By calculating the first and second derivatives of Equation (5), the angular velocities and angular accelerations of the joints can be expressed as

(7) $$\left\{\begin{array}{l} {\dot{\theta }}_{ij}=\left.f_{1}\left(V_{t},W_{t}\right)\right|V_{t}=\left(v_{tx},v_{ty},v_{tz}\right),W_{t}=\left(\dot{\alpha },\dot{\beta },\dot{\gamma }\right)\\ {\ddot{\theta }}_{ij}=\left.f_{1}\left(V_{t},W_{t},A_{t},T_{t}\right)\right|A_{t}=\left(a_{tx},a_{ty},a_{tz}\right),T_{t}=\left(\ddot{\alpha },\ddot{\beta },\ddot{\gamma }\right) \end{array}\right.,$$

where ***V****_t_* and ***W****_t_* are the velocity and the angular velocity of the trunk, respectively; ***A****_t_* and ***T****_t_* are the acceleration and the angular acceleration of the trunk, respectively.

By calculating the first and second derivatives of Equation (6), the velocity and the acceleration of the joint points and centers of mass of the links can be obtained as

(8) $$V_{ij}=\sum_{j=1}^{6}\left(a_{ij}\theta_{ij}+b_{ij}{\dot{\theta }}_{ij}\right)=\sum_{j=1}^{6}\left(a_{ij}F\left(\alpha ,\beta ,\gamma ,x,y,z\right)+b_{ij}\dot{F}\left(\alpha ,\beta ,\gamma ,x,y,z\right)\right)$$

(9) $$A_{ij}=\sum_{j=1}^{6}\left(c_{ij}\theta_{ij}+d_{ij}{\dot{\theta }}_{ij}+e_{ij}{\ddot{\theta }}_{ij}\right)=\sum_{j=1}^{6}\left(c_{ij}F\left(\alpha ,\beta ,\gamma ,x,y,z\right)+d_{ij}\dot{F}\left(\alpha ,\beta ,\gamma ,x,y,z\right)+e_{ij}\ddot{F}\left(\alpha ,\beta ,\gamma ,x,y,z\right)\right)$$

On this basis, the angular velocity and the angular acceleration of each link can be obtained by

(10) $$V_{i,j+1}=V_{i,j}+\omega_{i}\times L_{i}$$

(11) $${\dot{V}}_{i,j+1}={\dot{V}}_{i,j}+{\dot{\omega }}_{i}\times L_{i}+\omega_{i}\times \left(\omega_{i}\times L_{i}\right)$$

where $V_{i,j}$ and ${\dot{V}}_{i,j}$ are the velocity and the acceleration of the *j*-th joint of the *i*-th link, respectively. $V_{i,j+1}$ and ${\dot{V}}_{i,j+1}$ are the velocity and the acceleration of the (*j* + 1)-th joint of the *i*-th link, respectively. $\omega_{i}$ and ${\dot{\omega }}_{i}$ are the angular velocity and the angular acceleration of the *i*-th link, respectively. ***L****_i_* is the direction vector of the *i*-th link.

The driving torques can be obtained by establishing the Lagrange dynamic equation. The total kinetic energy of the robot can be expressed as

(12) $$E_{k}=\sum_{i=1}^{2}\sum_{j=1}^{2}\left(\frac{1}{2}{\;}^{0}V{{}_{ij}}^{T}m_{ij}{\;}^{0}V{}_{ij}+\frac{1}{2}{\;}^{0}\omega {{}_{ij}}^{T}{\;}^{0}I{}_{ij}{\;}^{0}\omega {}_{ij}\right)+\left(\frac{1}{2}{\;}^{0}V{{}_{t}}^{T}m_{t}{\;}^{0}V{}_{t}+\frac{1}{2}{\;}^{0}\omega {{}_{t}}^{T}{\;}^{0}I{}_{t}{\;}^{0}\omega {}_{t}\right),$$

where *m_ij_* and *m_t_* are the masses of the *j*-th link of the *i*-th leg and the trunk, respectively. ^0^*V_ij_* and ^0^*ω_ij_* are the velocity and the angular velocity of the *j*-th link of the *i*-th leg in the fixed coordinate system, respectively. ^0^*V_t_* and ^0^*ω_t_* are the velocity and the angular velocity of the trunk in the fixed coordinate system, respectively. ^0^*I_ij_* and ^0^*I_t_* are the moment of inertia in the fixed coordinate system, which can be expressed as

(13) $${\;}^{0}I{}_{ij(t)}={\;}^{0}T{}_{ij(t)}{\;}^{\mathrm{ij}}I{}_{ij(t)}{\;}^{0}T{{}_{ij(t)}}^{T},$$

where ^0^*T_ij_*_(*t*)_ is the transformation matrix of the *j*-th link of the *i*-th leg (or the trunk) in the fixed coordinate system. The total potential energy of the robot can be expressed as

(14) $$E_{p}=\sum_{i=1}^{2}\sum_{j=1}^{2}\left(m_{ij}gh_{ij}\right)+m_{t}gh_{t},$$

where *h_ij_* and *h_t_* are the distances from the center of mass of the link and the trunk to the ground, respectively, which can be obtained by kinematic analysis.

The trunk of the robot has 6 DOFs when two legs land at the same time. The drives are installed at the hip and knee joints of the legs because the ball pair formed by the contact between the legs and the ground is passive motion. At this time, the Lagrange dynamic equation can be written as

(15) $$\tau_{ij}=\frac{d}{dt}\frac{\partial E_{k}}{\partial {\dot{q}}_{ij}}-\frac{\partial E_{k}}{\partial q_{ij}}+\frac{\partial E_{p}}{\partial q_{ij}},$$

where $q_{ij}=\left(\theta_{i1},\theta_{i2},\theta_{i3},\theta_{i4},\theta_{i5},\theta_{i6}\right)$, and ${\dot{q}}_{ij}=\left({\dot{\theta }}_{i1},{\dot{\theta }}_{i2},{\dot{\theta }}_{i3},{\dot{\theta }}_{i4},{\dot{\theta }}_{i5},{\dot{\theta }}_{i6}\right)$.

Through the method detailed above, the dynamic model of the cheetah-inspired quadruped robot moving at a high speed in 3D space can be established.

### 2.3. Establishment of Stability Index System

On the basis of establishing the dynamic model, the stability index system must be set up so that the robot has good dynamic performance by optimizing the leg parameters. A two-level stability index system is proposed, including a minimum index system and range index system.

The indices contained in the minimum index system should be as small as possible during robot movement. It includes the total inertia moment, the angular velocity of the trunk, the zero moment point (ZMP), and the energy consumption of the robot in a motion cycle.

(1) Total inertia moment. During the high-speed movement of the robot, it should maintain good stability without overturning and rolling over. During the descending and ascending phases of the trunk, the mean and the variance of the total inertia moment and the total inertia moment of the robot at the moment of leaving the ground should be as small as possible. The total inertia moment at the *k*-th time can be expressed as

(16) $$M_{Ik}=\sum_{i=1}^{2}\sum_{j=1}^{2}\left(r_{ijk}\times F_{ijk}+M_{ijk}\right)+r_{tk}\times F_{tk}+M_{tk},$$

where ***F****_ij_* and ***F****_t_* are the inertia forces of the *j*-th link of the *i*-th leg and the trunk, respectively. ***r****_ij_* and ***r****_t_* are the vectors of the center of mass of the *j*-th link of the *i*-th leg and the trunk in the fixed coordinate system, respectively. ***M****_ij_* and ***M****_t_* are the inertia moments. The above indices can be expressed as

(17) $$\left\{\begin{array}{l} D=\left|\forall D\left(M_{ij(t)}\right)-\forall D\left(M_{kp}\right)\right|\\ E=\left|\forall E\left(M_{ij(t)}\right)\right|\\ V=End\left(M_{ij(t)}\right) \end{array}\right.,$$

where *D*, *E*, and *V* represent the mean, the variance, and the end value, respectively.

(2) Angular velocity of the trunk. A small total inertia moment can make the robot have a small angular acceleration, but further ensuring that the trunk has a small angular velocity at the moment of leaving the ground is still necessary to prevent the robot from turning during a long flight time. The angular velocity of the robot can be obtained according to Equations (10)–(11).

(18) $$\left(\omega_{r},\alpha_{r}\right)=g\left(\theta_{ij},\dot{\Phi },\ddot{\Phi }\right)$$

(3) ZMP. ZMP can be expressed as [42]

(19) $$\left\{\begin{array}{l} X_{ZMP}=\frac{\sum_{i=1}^{4}m_{ij}\left({\ddot{y}}_{ij}+g\right)x_{ij}+m_{t}\left({\ddot{y}}_{t}+g\right)x_{t}-\sum_{i=1}^{4}m_{ij}{\ddot{x}}_{ij}y_{ij}-m_{t}{\ddot{x}}_{t}y_{t}}{\sum_{i=1}^{4}m_{i}\left({\ddot{y}}_{ij}+g\right)+m_{t}\left({\ddot{y}}_{t}+g\right)}\\ Y_{ZMP}=0\\ Z_{ZMP}=\frac{\sum_{i=1}^{4}m_{ij}\left({\ddot{y}}_{ij}+g\right)z_{ij}+m_{t}\left({\ddot{y}}_{t}+g\right)z_{t}-\sum_{i=1}^{4}m_{ij}{\ddot{z}}_{ij}y_{ij}-m_{t}{\ddot{z}}_{t}y_{t}}{\sum_{i=1}^{4}m_{i}\left({\ddot{y}}_{ij}+g\right)+m_{t}\left({\ddot{y}}_{t}+g\right)} \end{array}\right.$$

where (*x_ij_*, *y_ij_*, *z_ij_*) and (*x_t_*, *y_t_*, *z_t_*) are the position coordinates of the center of mass of the *j*-th link of the *i*-th leg and the trunk in the fixed coordinate system, respectively.

(4) Energy consumption. The energy consumption of the robot in high-speed motion should be as small as possible. The total energy consumption can be expressed as

(20) $$C=\int_{0}^{T}\sum_{i=1}^{2}\sum_{j=1}^{3}\left|P_{ij}\left(t\right)\right|dt=\int_{0}^{T}\sum_{i=1}^{2}\sum_{j=1}^{3}\left|\tau_{ij}\left(t\right)\omega_{ij}\left(t\right)\right|dt,\;$$

where *P_ij_* is the instantaneous power of the *j*-th joint of the *i*-th leg, *τ_ij_* is the joint torque, and *ω_ij_* is the joint angular velocity.

The indices contained in the range index system are considered to meet the requirements within given ranges. This includes the driving torques of the legs and the leg swing angle.

(1) Driving torques. The mean value of the discrete points of the driving torques for different joints should be in a small range. In this way, motors with the same model can be selected, reducing the difficulty of robot prototype development and control. The variances of the joint torque should also be in a small range to ensure the smoothness of torque changes, and prevent excessive torque changes from affecting the service life of the motor. The above indices can be expressed as

(21) $$\left\{\begin{array}{l} D=\left|\forall D\left(\tau_{ij}\right)-\forall D\left(\tau_{kp}\right)\right|\\ E=\left|\forall E\left(\tau_{ij}\right)\right| \end{array}\right.,$$

where *D* and *E* represent the mean and the variance, respectively.

(2) Leg swing angle. The leg swing angle refers to the angle between the line between the hip joint and the landing point and the vertical direction. If the leg swing angle is too large, the robot easily loses stability due to small friction. Therefore, the leg swing angle should be smaller than the given values. The leg swing angle can be expressed as

(22) $$\Psi =\left(\arccos\frac{z\left(R_{i6}\right)-z\left(R_{i1}\right)}{y\left(R_{i6}\right)-y\left(R_{i1}\right)}\right),$$

where *R_ij_* is the position vector of the *j*-th joint of the *i*-th leg.

Through the above analysis, a two-level stability index system including the minimum index system and the range index system is established. Among them, the constraints for total inertia moment, angular velocity of the trunk, ZMP, and leg swing angle determine the feasibility of robot motion, and the constraints for energy consumption and driving torques determine the performance advantages of robot long-term movement. The stability index system provides a basis for the subsequent optimization of the motion parameters of the robot.

### 2.4. Leg parameter Optimization Method

According to the analysis results of the biological mechanism in Section 2.1, the leg postures and the driving torques of the robot during high-speed movement must be determined, which can be obtained through optimization.

The optimization variables include leg posture parameters and trunk motion parameters. The former includes *h*_1*i*_, *h*_2*i*_, *d_ki_*, and *φ_ki_* (*I* = 1, 2), as shown in Figure 2b. The latter includes the polynomial coefficients shown in Equations (1) and (3). In particular, the polynomial coefficients are different in the descending and ascending phases of the trunk.

The optimization objective function can be expressed as

(23) $$\begin{array}{l} Z=Min\sum_{i=1}^{n}w_{i}f_{i}\left(x_{1},\ldots ,x_{n}\right)\\ s.t.\Gamma \end{array}$$

where

$$\begin{array}{l} f_{1}=\left(D\left|M_{Ik}\right|-min\left(D\left|M_{Ik}\right|\right)\right)/\left(max\left(D\left|M_{Ik}\right|\right)-min\left(D\left|M_{Ik}\right|\right)\right)\\ f_{2}=\left(E\left|M_{Ik}\right|-\min\left(E\left|M_{Ik}\right|\right)\right)/\left(max\left(E\left|M_{Ik}\right|\right)-\min\left(E\left|M_{Ik}\right|\right)\right)\\ f_{3}=\left(End\left(\left|M_{Ik}\right|\right)-min\left(\left|M_{Ik}\right|\right)\right)/\left(max\left(\left|M_{Ik}\right|\right)-min\left(\left|M_{Ik}\right|\right)\right)\\ f_{4}=\left(\left|D\left(ZMP,A_{i}A_{i+1}\right)\right|-\min\left|D\left(ZMP,A_{i}A_{i+1}\right)\right|\right)/max\left(\left|D\left(ZMP,A_{i}A_{i+1}\right)\right|-\min\left|D\left(ZMP,A_{i}A_{i+1}\right)\right|\right)\\ f_{5}=\left(\left|E\left(ZMP,A_{i}A_{i+1}\right)\right|-\min\left|E\left(ZMP,A_{i}A_{i+1}\right)\right|\right)/max\left(\left|E\left(ZMP,A_{i}A_{i+1}\right)\right|-\min\left|E\left(ZMP,A_{i}A_{i+1}\right)\right|\right)\\ f_{6}=\left(C-\min C\right)/\left(\max C-\min C\right) \end{array}$$

*f*_1_ and *f*_2_ represent the mean and variance of the discrete points of the total inertia moment of the robot in the descending and ascending phases, respectively. *f*_3_ represents the total inertia moment of the robot at the moment when it leaves the ground. *f*_4_ and *f*_5_ represent the mean and the variance of the distance from the ZMP to the line between the two landing points, respectively. *f*_6_ represents the energy consumption. In particular, Equation (3) is derived, and the zero angular velocity of the trunk at the end of the descending and ascending phases can be taken as the boundary condition instead of being listed as the objective function. The relationship between polynomial coefficients and time can be obtained and used as the constraints for optimization. This can ensure that the angular velocity of the trunk of the robot is zero when it leaves the ground, and that the robot has good stability. *f_i_* (*i* = 1, 2,…, 6) should be as small as possible, which corresponds to the minimum index system. *w_i_* is the weight coefficient, which is determined by analytic hierarchy process (AHP). The weight coefficient can be expressed as

(24) $$w_{i}=\frac{w_{j}^{0}}{\sum_{j=1}^{n}w_{j}^{0}},$$

where $w_{i}^{0}$ are the values obtained by adding rows after the standardization of the judgment matrix. In particular, the consistency of the judgment matrix must be checked to ensure that the scoring of experts is logical and does not appear contradictory. Consistency index can be expressed as

(25) $$CR=\frac{\lambda_{max}-n/n-1}{RI},\;$$

where *λ*_max_ is the maximum eigenvalue of the judgement matrix, and *RI* is an average random consistency index, which can be obtained by looking up the table.

For Equation (23), ***Γ*** is the constraints, which can be expressed as

(26) $$\left\{\begin{array}{l} \left|\forall D\left(\tau_{ij}\right)-\forall D\left(\tau_{kp}\right)\right|\leq Z_{D}and\left|\forall E\left(\tau_{ij}\right)\right|\leq Z_{E}\\ \Psi \leq \Psi_{o} \end{array}\right.,$$

where the first formula indicates that the mean and the variance of the driving torques should meet the range requirements, and *Z_D_* and *Z_E_* are the given reference values. The second formula indicates that the angle between the leg and the ground should be less than the given value **Ψ**_0_. Equation (26) is consistent with the range index system.

For the above optimization variables and objective functions, an improved bee colony algorithm is applied in this paper, and the optimization is shown in Algorithm 1. First, the initial ranges of optimization variables ***Q***, the kinematic feasible region ***O*** (make sure the trunk is in the workspace), and the maximum value ***Z***(*i*)*_max_* and minimum value ***Z***(*i*)*_min_* of the objective function are given. A set of optimization variables ***W***(*j*) is taken from the initial range ***Q****^range^*, and each item in the optimization objective function ***Z***(*j*) and the motion parameters ***O***(*j*) are calculated based on bee colony algorithm $H_{rule}^{G}$ and range constraints ***M***. By judging the value of the objective function, ***Q****^range^*, ***Z***(*i*)*^min^*, and ***Z***(*i*)*^max^* are updated and assigned to $Q_{new}^{range}$,$Z{\left(\mathrm{(i)}\right)}_{new}^{\min}$, and $Z{\left(i\right)}_{new}^{\max}$, respectively, to obtain the approximate accurate values. While carrying out the accurate dimensionless processing of the objective function, the design efficiency is improved through the accurate constraints of the ranges. On this basis, values are taken from $Q_{new}^{range}$, the objective function ***Z***(*j*) is calculated, and $Q_{new}^{range}$,$Z{\left(i\right)}_{new}^{\min}$, and $Z{\left(i\right)}_{new}^{\max}$ are simultaneously updated to improve the constraint accuracy continuously. In particular, when the ratio of the total number of cycles to the current number of cycles is a positive natural number, the result obtained by the previous generation calculation is used as a reference to reduce the ranges by multiplying the scale coefficient *k* (*k* < 1) to improve the calculation efficiency. Finally, the minimum value of the objective function is obtained, and the optimization variables are output.
**Algorithm 1 Leg parameters optimization**Nomenclature:***Q***% Ranges of optimization variables
***W***% Optimization variables
***Z***% Optimization objective function
***O***% Kinematic feasibility constraints
***M***% Range constraints
$H_{rule}^{G}$% Bee colony algorithm1: Set ***Q****^range^*, ***O****^range^*, ***Z***(*i*)*^min^*,***Z***(*i*)*^max^*, and ***M***2:**For***j*=1, …., *N_1_*3:      Select ***W***(*j*) from ***Q****^range^*4:$\mathrm{Calculate}\;Z(j)\;\mathrm{and}\;(j)\;\mathrm{by}\;H_{rule}^{G}$ and***M***5:   **If *O***(*j*) ∈***O**^range^*6:    Update ***Q**^range^*,***Z***(*i*)*^min^* and ***Z***(*i*)*^max^ by **Z***(*j*)7:   $Q_{new}^{range}$ = ***Q**^range^*
$,\;Z{\left(i\right)}_{new}^{\min}$
***Z***(*i*)*^min^* and $Z{\left(i\right)}_{new}^{\max}$ = *Z*(*i*)*^max^*8:**End If**9:**End For**10:**For***j* = 1, …., *N*_2_11:$\mathrm{Select}\;(j)\;\mathrm{from}Q_{new}^{range}$12:$\mathrm{Calculate}\;Z(j)\;\mathrm{by}H_{rule}^{G}$ and ***M***13:${\mathrm{Update}}_{\;}Q_{new}^{range}$$,Z{\left(i\right)}_{new}^{\min}$$\;\mathrm{and}\;Z{\left(i\right)}_{new}^{\max}$*by **Z***(*j*)14:**If** *N_2_*/j∈N^*^15:$\mathrm{Update}\;Q_{new}^{range}$16:**End if**17:$\mathrm{Calculate}\;Z\;\mathrm{by}\;Z{\left(i\right)}_{new}^{\min}$$\;\mathrm{and}\;Z{\left(i\right)}_{new}^{\max}$18:If ***Z*** ≤ ***Z****_best_*19:               Copy *Z* into *Z_best_*20:**End if**21:**End For**

In particular, for the above optimization process, parallel calculation is used in the process of employed bees, on-looker bees, and scout bees to find honey sources, and the extreme value of the objective function is dynamically updated after the calculation for each kind of bee is completed. At the same time, the dynamic parameters for the scout bees are added, and the working threshold of the scout bees is adjusted dynamically according to the convergence of the objective function. The above process can improve the convergence speed and enhances the ability of global optimal search.

## 3. Results

### 3.1. Examples

To prove the feasibility of the method proposed in this paper, two examples are given. The structural parameters of the robot are shown in Table 1. The variable (*a*_2_, *b*_2_) represents the coordinates of the hip joint in the moving coordinate system *O_t_*–*X_t_Y_t_Z_t_*, as shown in Figure 2b. The thigh and the calf legs are cylinders, and the section radius *r* and length *h* are given in Table 1. For example 1, the known parameters are shown in Table 2. *v*_1_ and *v*_2_ are the velocities of the trunk at points *O*_1_ and *O*_3_, respectively; (*x*_1_, *y*_1_, *z*_1_) and (*x*_2_, *y*_2_, *z*_2_) are the coordinates of points *O*_1_ and *O*_2_, respectively. **Φ**_0_ is the trunk posture angle at the moment of landing. **Ψ**_0_, *Z_D_*, and *Z_E_* are the given values shown in Equation (26).

To ensure the motion stability of the robot, the trunk movement trajectory and posture change rule are assumed cubic functions, and the polynomial coefficients need to be determined. In the descending phase of the trunk, the trajectory equation and the posture equation of the trunk have 12 undetermined polynomial coefficients. The position and the velocity of the trunk at *O*_1_ and *O*_2_ are known, the angle and angular velocity of the trunk at *O*_1_ and *O*_2_ are known, and the trunk does not rotate around the *Z_t_* axis. By substituting the boundary conditions into Equations (1) and (3), all polynomial coefficients can be expressed as functions of time. In the ascending phase of the trunk, the position and the velocity of the trunk at *O*_2_, the velocity direction of the trunk at *O*_3_, the trunk angle and the angular velocity at *O*_2_, and the trunk angular velocity at *O*_3_ are known. The relationship between the undetermined coefficients and the movement time can also be obtained by substituting the boundary conditions into Equations (1) and (3). However, not all polynomial coefficients can be expressed in time. Two coefficients in Equation (1) and three coefficients in Equation (3) still need to be determined. To sum up, the optimization variables involved in the trunk motion to be determined include *t*_1_ (movement time of trunk in the descending phase), *t*_2_ (movement time of trunk in the ascending phase), *a*_12_, *a*_22_, *d*_12_, *d*_22_, and *d*_32_. The optimization variables also include leg landing point parameters *h*_1*i*_, *h*_2*i*_, *d_ki_*, and *φ_ki_*. The initial ranges of the optimization variables are listed randomly in Table 3. The weight coefficients are *w*_1_ = 0.0755, *w*_2_ = 0.0464, *w*_3_ = 0.5984, *w*_4_ = 0.1305, *w*_5_ = 0.0623, and *w*_6_ = 0.0869. For the hierarchical bee colony algorithm, the number of honey sources is 100, the number of leading bees is 100, and the maximum number of iterations is 100. The optimization results obtained by the method proposed in this paper are shown in Table 3.

**Figure 3:**
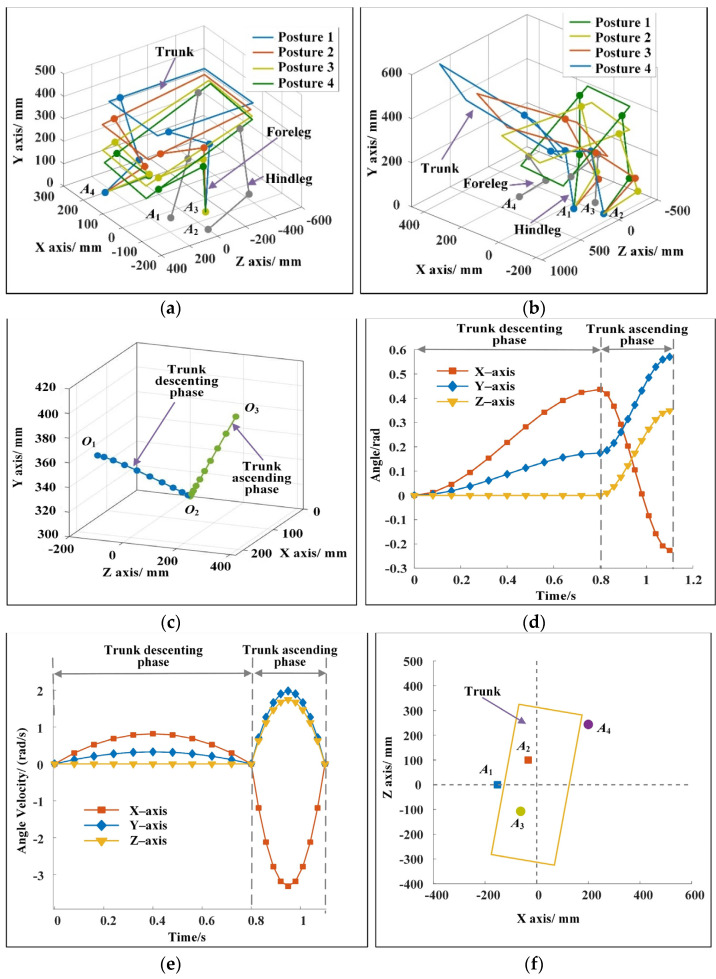
(**a**) Motion sequence of the robot in the descending phase of the trunk for example 1. (**b**) Motion sequence of the robot in the ascending phase of the trunk for example 1. (**c**) Movement trajectory of the trunk for example 1. In the descending and ascending phases of the trunk, the trajectories are cubic functions. (**d**) Changes of trunk posture for example 1. In the descending phase of the trunk, the angle changes of the trunk around the three axes are 25°, 10°, and 0°. In the ascending phase of the trunk, the angle changes of the trunk around the three axes are 38.01°, −22.69°, and −20.00°. (**e**) Changes of trunk angular velocity for example 1. The angular velocity of the robot at the moment of leaving the ground is zero. (**f**) Landing points of the forelegs and the hindlegs for example 1.

Figure 3a,b show the motion sequence when the robot changes motion direction during running. In the descending phase of the trunk, the two forelegs of the robot are in contact with the ground, and the center of mass of the trunk moves 243.72 mm in 0.8 s. In the ascending phase of the trunk, the two hindlegs of the robot are in contact with the ground, and the center of mass of the trunk moves 492.58 mm in 0.3 s. Figure 3c shows the trajectory of the center of mass of the trunk. When the forelegs of the robot touch the ground, the motion direction vector of the trunk is (0, 0, 1). At the moment when the hindlegs of the robot leave the ground, the motion direction vector of the trunk is (1.74, 0.5, 1.84). From the top view, the included angle of the direction vector is 28.08°, and the running direction of the robot changes clearly. Figure 3d shows the change of trunk posture. The proper change of body posture is conducive to keeping the good dynamic stability of the robot. The angles of the robot around the three axes at the moment of leaving the ground are −13.01°, 32.69°, and 19.81°. The changes of angular velocities obtained by the method described in Section 2.4 are shown in Figure 3e. The angular velocity of the robot at the moment of leaving the ground is zero, and the trunk of the robot will not rotate significantly in the flight phase. Figure 3f shows the positions of the landing points of the legs. In the descending phase of the trunk, the coordinates of the landing point of the two forelegs are (200, 0, and 243.81 mm) and (−62.01, 0, and −107.17 mm). In the ascending phase of the trunk, the landing point coordinates of the two hindlegs are (−151.93, 0, and 0.04 mm) and (−32.88, 0, and 99.98 mm). The leg landing points are no longer symmetrical along the *Z*_0_ axis, and the legs have evident adduction/abduction angles. The maximum leg swing angles of the forelegs are 41.04° and 41.09°, and the maximum leg swing angles of the hindlegs are 45.09° and 41.00°. This outcome is consistent with the analysis results of the movement mechanism of the cheetah when it turns during running, as shown in Figure 1a.

**Figure 4:**
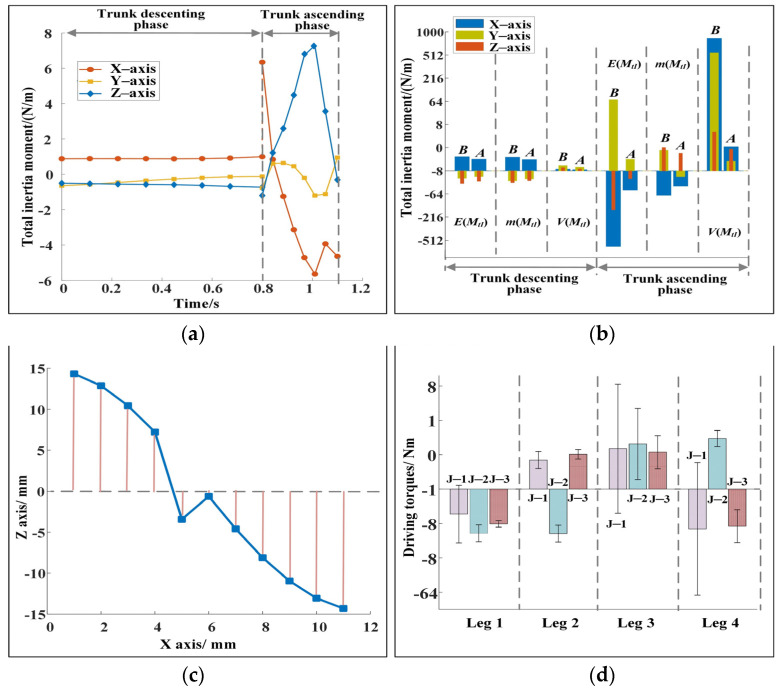
(**a**) Change of the total inertia moment of the robot in the descending and the ascending phases after optimization for example 1. (**b**) Changes of the total inertia moment of the robot before and after optimization for example 1. The maximum reduction of the end value, the mean, and the variance of the total inertia moment of the robot after optimization are 99.6%, 97.7%, and 99.9%, respectively, and the decreases are evident. (**c**) Change of ZMP during the descending phase of the trunk for example 1. ZMP changes near the connecting line of the landing points of two legs, showing good stability. (**d**) Mean and variance of the driving torques for example 1. The maximum difference of the mean value is 2.8 N·m, and the maximum variance is 7.16 N·m. The driving torques are within reasonable ranges.

The dynamic performance of the cheetah-inspired quadruped robot during steering is shown in Figure 4. The change of the total inertia moment of the robot is shown in Figure 4a. In the descending phase of the trunk, the amplitude of the total inertia moment of the robot is small and changes gently. When the trunk reaches the lowest point *O*_2_, the total inertia moments of the robot along the three axes are 0.99, −0.12, and −0.73 N·m. In the ascending phase of the trunk, the total inertia moment increases substantially but then decreases rapidly because the robot needs to obtain a large acceleration in a short time. When the trunk reaches point *O*_3_, the total inertia moments of the robot along the three axes are −4.64, 0.94, and −0.31 N·m. Figure 4b shows the changes of the total inertia moment before and after optimization. “*B*” and “*A*” refer to before and after optimization, respectively. *E*(*M_tI_*), *m*(*M_tI_*), and *V*(*M_tI_*) represent the end value, the mean, and the variance of the total inertia moment, respectively. The total inertia moment before optimization is calculated by substituting the initial parameters. Figure 4b shows that the total inertia moment decreases considerably after optimization. In the descending phase of the trunk, the maximum reductions of *E*(*M_tI_*), *m*(*M_tI_*), and *V*(*M_tI_*) after optimization are 54.22%, 47.79%, and 78.96%, respectively. In the ascending phase of the trunk, the maximum reductions of *E*(*M_tI_*), *m*(*M_tI_*), and *V*(*M_tI_*) after optimization are 99.6%, 97.7%, and 99.9%, respectively. The dynamic stability of the robot is remarkably improved. Figure 4c shows the distance from ZMP to the connecting line between the two landing points during the descending phase of the trunk. The average value of the distance is 9.08 mm. ZMP is near the connecting line of two points. The deviation is small compared with the size of the robot, and the robot has good dynamic stability. Figure 4d shows the mean and variance of driving torques. “J-1,” “J-2,” and “J-3” represent hinges 1, 2, and 3, respectively, as shown in Figure 1b. The maximum difference between the mean values of driving torques of the different joints is only 2.8 N/m, and the driving torques change smoothly with a slight difference in amplitude. Moreover, the energy consumption of the robot during movement is 27.96 J. The above analysis results reveal that the optimized indices that are contained in the minimum index system are very small, and the indices contained in the range index system are within reasonable ranges. The robot has good dynamic stability by using the parameters of the leg postures and the driving torques obtained by the method proposed in this paper.

For example 1, the robot turns left during running from the top view, thus the projection of the motion direction vector on the ground of the robot at the moment of leaving the ground is counterclockwise relative to that of the robot at the moment of landing. To prove the feasibility of the method proposed in this paper further, an example of the robot turning to the right is given. The known values remain unchanged, as shown in Table 2. The initial range of optimization variables and optimization results are shown in Table 4 and Table 5.

**Figure 5:**
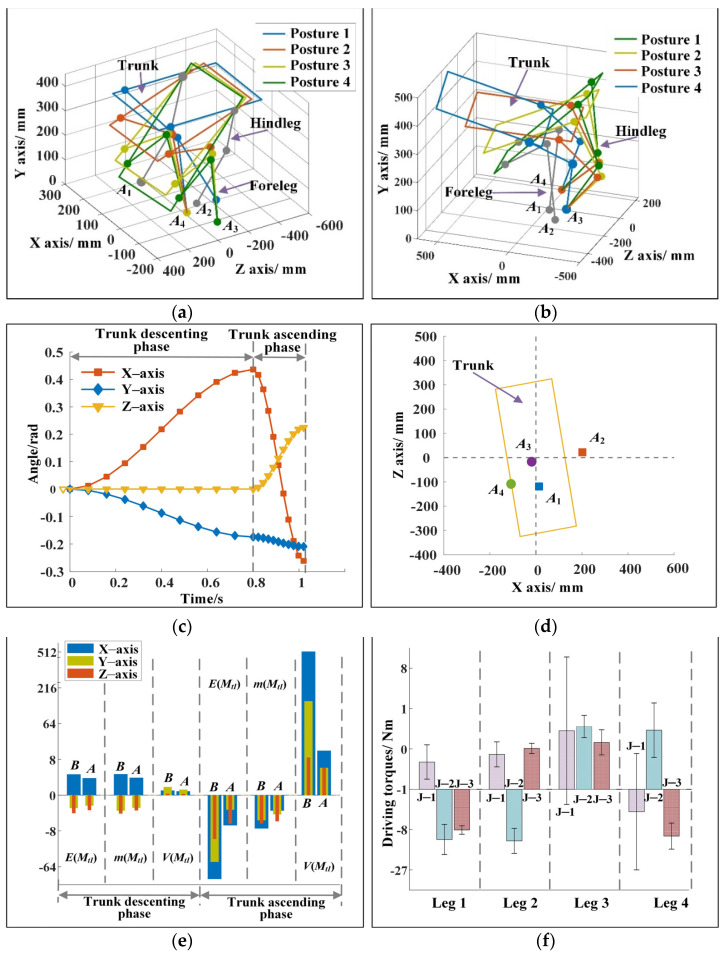
(**a**) Motion sequence of the robot in the descending phase of the trunk for example 2. (**b**) Motion sequence of the robot in the ascending phase of the trunk for example 2. (**c**) Changes of trunk posture for example 2. In the descending phase of the trunk, the angle changes of the trunk around the three axes are 25°, −10°, and 0°. In the ascending phase of the trunk, the angle changes of the trunk around the three axes are −40.01°, −12°, and 12.88°. (**d**) The landing points of the forelegs and hindlegs for example 2. (**e**) Changes of the total inertia moment of the robot before and after optimization for example 2. The maximum reductions of the end value, the mean, and the variance of the total inertia moment of the robot after optimization are 98.9%, 90.2%, and 97.6%, respectively, and the decreases are evident. (**f**) Mean and variance of the driving torques for example 2. The maximum difference of the mean value is 5.88 N·m, and the maximum variance is 6.12 N·m. The driving torques are within reasonable ranges.

The optimization results for example 2 are shown in Figure 5. Figure 5a,b show the motion sequence of the robot for example 2. In the descending phase of the trunk, the center of mass of the trunk moves 243.72 mm in 0.8 s. In the ascending phase of the trunk, the center of mass of the trunk moves 337.3 mm in 0.22 s. Figure 5c shows the change of trunk posture. Similarly, the angular velocity of the trunk at the moment of leaving the ground is zero. Figure 5d shows the positions of the landing points of the legs. The maximum leg swing angles of the forelegs and hindlegs are (53.30°, 48.10°) and (33.75°, 27.71°), respectively. The dynamic performance of the cheetah-inspired quadruped robot during steering is shown in Figure 5e,f. Figure 5e shows the changes of the total inertia moment before and after optimization. Similarly, the total inertia moment before optimization is calculated by substituting the initial parameters. Compared with those before optimization, in the descending phase of the trunk, the maximum reductions of *E*(*M_tI_*), *m*(*M_tI_*), and *V*(*M_tI_*) after optimization are 45.8%, 46.9%, and 68.8%, respectively. In the ascending phase of the trunk, the maximum reductions of *E*(*M_tI_*), *m*(*M_tI_*), and *V*(*M_tI_*) after optimization are 98.89%, 90.2%, and 97.57%, respectively. Moreover, the total inertia moments of the robot around the three axes at the moment of leaving the ground are −4.7069, −0.5166, and −3.9464 N·m within small ranges. This finding shows that the robot has good dynamic stability. Figure 5f shows the mean and the variance of the driving torques. The maximum difference between the mean values of driving torques of the different joints is only 5.88 N/m, and the variance is not too large.

### 3.2. Simulation

**Figure 6:**
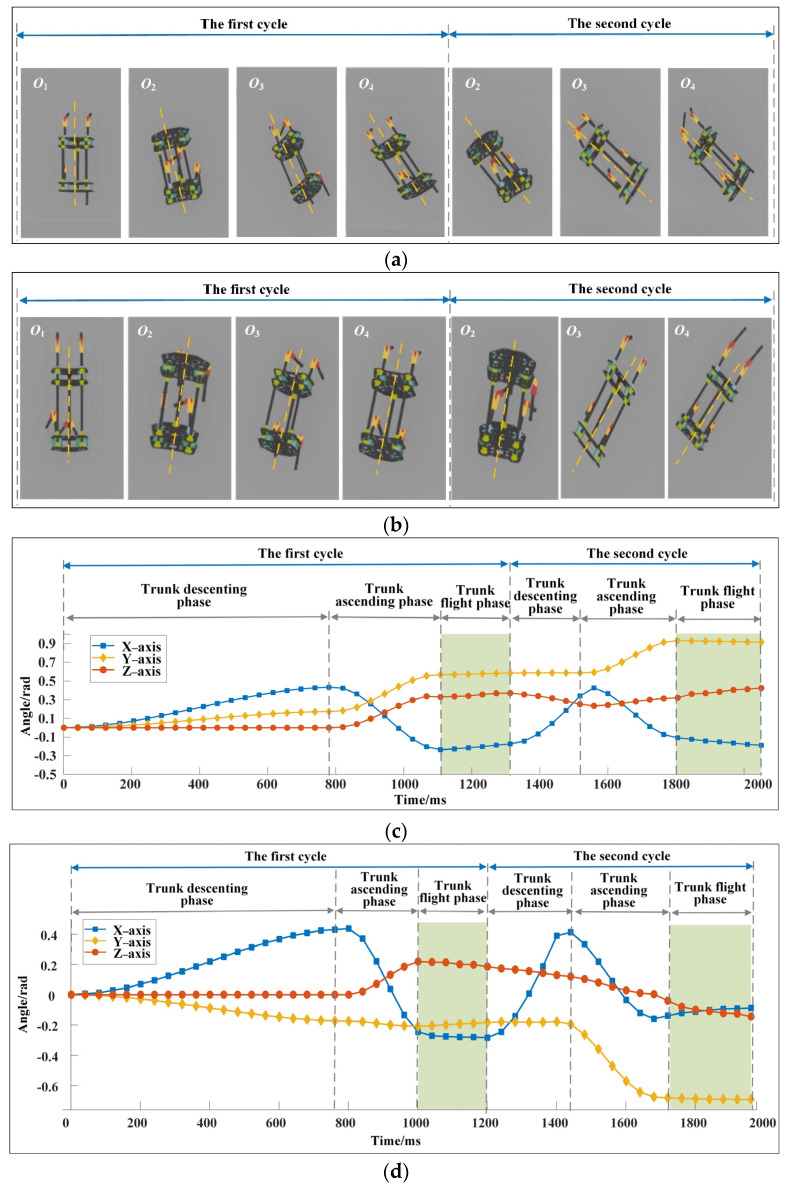

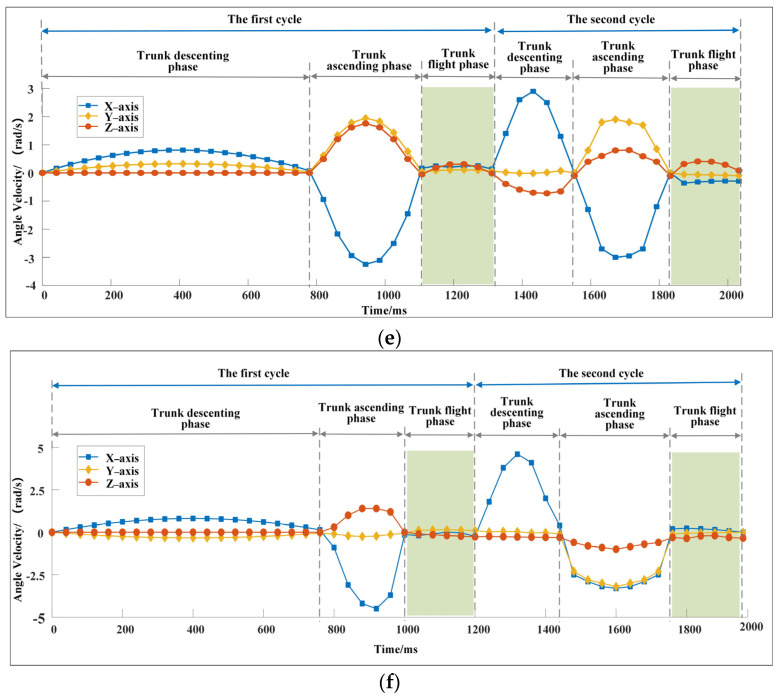
(**a**) Top view of the motion sequences of the robot for simulation example 1. The robot rotates 28.08° around the vertical axis in both cycles, and the direction of motion changes considerably. (**b**) Top view of the motion sequences of the robot for simulation example 2. The robot rotates −28.28° around the vertical axis in both cycles. (**c**) Change of trunk posture for simulation example 1. The changes of the maximum rotation angles of the trunk in the flight phase are −2.95°, 3.75°, and 3.32°. (**d**) The change of trunk posture for simulation example 2. The changes of the maximum rotation angles of the trunk in the flight phase are 1.88°, 0.92°, and −3.72°. (**e**) Change of trunk angular velocity of the trunk for simulation example 1. (**f**) Change of trunk angular velocity of the trunk for simulation example 2.

Two examples are simulated with Webots to verify that the robot can turn quickly while running and has good dynamic stability, and the simulation videos are shown in Supplementary Materials. Each example contains two continuous running cycles. The structural parameters of the robot are consistent with theoretical calculation. Since the robot has a long flight time during running, the stability of the robot is directly reflected by the rotation angle of its trunk. For simulation example 1, the robot turns left continuously while running. Taking the joint angles and driving torques obtained by theoretical calculation as input for the first running cycle, the input parameters of the robot in the second running cycle can be calculated by the same method. The top view of the motion sequences of the robot in two cycles is shown in Figure 6a. *O*_1_, *O*_2_ and *O*_3_ refer to the position of the center of mass of the trunk shown in Figure 2a, and *O*_4_ refers to the highest point of the robot in the flight phase. The robot rotates 28.08° around the vertical axis in both cycles, and the motion direction changes substantially. The trunk rotation angles corresponding to Figure 6a is shown in Figure 6c. In the descending and ascending phases of the trunk of the robot in the first running cycle, the trunk rotation angles are exactly the same as those shown in Figure 3d. From one perspective, it proves the correctness of theoretical calculation; conversely, it can also show that the robot moves according to the predetermined rules in the descending and ascending phases of trunk, without movement failure, such as rollover. In the flight phase, the trunk posture of the robot almost remains unchanged. The maximum rotation angles of the trunk in two cycles around the three axes are −2.95°, 3.75°, and 3.32°. This finding shows that the robot has small angular velocity at the moment of leaving the ground, which can be seen in Figure 6e, and it also proves the correctness of the proposed two-level stability index system. For simulation example 2, the top views of the motion sequences of the robot and the trunk rotation angle are shown in Figure 6b,d, respectively. The robot rotates −28.08° around the vertical axis in both cycles. In the descending and ascending phases of the trunk of the robot in the first cycle, the trunk rotation angles are exactly the same as those shown in Figure 4c. In the flight phase, the maximum rotation angles of the trunk in two cycles around the three axes are 1.88°, 0.92°, and −3.72°. The change of the angular velocity of the trunk corresponding to simulation example 2 is shown in Figure 6f, and the angular velocity of the robot at the moment of leaving the ground in two cycles is approximately zero. The rotation angles of the trunk in the flight phases and the angular velocity of the trunk at the moment of leaving the ground are within small ranges, and the robot shows good dynamic stability.

The above simulation results show that the proposed method in this paper can make the trunk posture of the robot stable and achieve good dynamic stability in high-speed steering motion by controlling the leg postures and the driving torques. The cheetah-inspired quadruped robot does not overturn or roll over due to excessive velocity and change of movement direction, so that the movement fails.

## 4. Discussion

In this paper, the main research objective is to propose a method to maintain the dynamic stability of the robot during steering running. Therefore, a two-level stability index system, including a minimum index system and a range index system, is proposed based on the dynamic model, and optimization objective functions are established based on the index system. The optimization variables include not only leg posture parameters, but also the trunk movement trajectory and posture parameters. Through the coordination of leg postures and driving torques obtained by the improved bee colony algorithm, the legged robot can achieve good dynamic performance [42,43,44]. The method proposed in this paper can make the quadruped robot achieve fast steering in running, and the following factors need to be considered.

**Figure 7:**
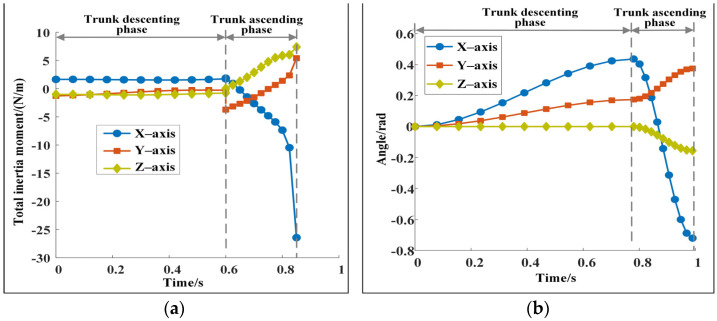
(**a**) Change of the total inertia moment when the trunk is forced horizontally. The maximum value of the total inertia moment is 27.97 N·m, which is much greater than the value shown in Figure 4a. (**b**) A possible trunk posture change rule. Although the corresponding total inertia moment is within a reasonable range, the maximum pitching angle of the trunk is 41.28°, which is not conducive to the stability of the robot.

(1) Changes in trunk posture. Figure 3 and Figure 5 show that the posture of the trunk changes during the descending and ascending phases. If the trunk is forced to remain horizontal without a posture change, although the trunk looks more stable, keeping the total inertia moment within a small range at the moment of the robot leaving the ground is difficult. The robot turns over evidently in the flight phase, which leads to motion failure. Figure 7a shows the change of the total inertia moment of the robot when the trunk is forced horizontally. The total inertia moments of the robot at the moment of leaving the ground are −26.43, 5.41, and 7.40 N·m. They are substantially larger than those shown in Figure 4 and Figure 5. Moreover, the change of the trunk angle should be reasonable. For example, Figure 7b shows a set of calculated results for trunk angle changes. Although the total inertia moment of the robot corresponding to Figure 7b is within a reasonable range and the robot is stable, the pitch angle of the robot at the moment of leaving the ground is 41.28°, which is not conducive to the stability of the robot in the next cycle. Figure 3d and Figure 5c show that the pitch angle of the trunk is relatively small at the moment of leaving the ground, and this is conducive to the robot maintaining good dynamic stability.

(2) Coupling of multiple parameters. High-speed motion in 3D space has many dynamic stability indices, and the coupling degree between indices is high. For example, a large coupling relationship exists between the steering angle, the velocity, the total inertia moment of the robot at the moment of leaving the ground, and ZMP. When the velocity of the robot at the moment of leaving the ground increases—that is, when the forward distance of the robot in a cycle increases—or the steering angle is too large, always staying near the connecting line the landing points of the two legs is difficult for the ZMP of the robot and the total inertia moment increases, thus the robot has difficulty maintaining good stability. For example, when the velocity of the trunk increases from 3.5 m/s to 5 m/s at the moment of leaving the ground, the total inertia moment after optimization remarkably increases from 6.16 N·m to 14.21 N·m. Although it can increase the movement time of the trunk in the ascending phase to reduce the total inertia moment, the difficulty of obtaining the optimal solution increases. Therefore, the motion parameters of the robot must be reasonably determined to achieve continuous, stable motion.

(3) Determination of weight coefficients. For the optimization objective function shown in Equation (23), the weight coefficients influence the results. For the examples shown in Section 3.1, the weight coefficients are determined by AHP. *f*_3_ and *f*_4_ have a great influence on dynamic stability, and their weight coefficients are large; *f*_1_, *f*_2_, *f*_5_, and *f*_6_ have minimal influence on dynamic stability, and their weight coefficients are relatively small. If the weight coefficients are changed to *w_i_*=1/*i*, the optimization results show that the total inertia moments of the robot after optimization at the moment of leaving the ground are −21.31, 4.35, and −5.29 N·m. The dynamic stability clearly deteriorates. Therefore, using experts’ experience to determine the importance of the indices to determine the weight coefficients is reasonable.

However, due to the complexity of the optimization objective functions and the large number of optimization variables, the current optimization efficiency cannot meet the real-time requirements. The robot needs to complete the motion planning in advance under the known terrain to achieve complex high-speed movement. In the future, on the basis of the method proposed in this paper, the data set can be established and the method to maintain the dynamic stability of the robot during steering running based on deep neural network can be further proposed. The efficiency of the algorithm will be further improved, making it possible for the robot to complete high-speed steering movement in real time.

## 5. Conclusions

The steering of the quadruped robot during high-speed running is of great importance for improving its movement flexibility. However, too many optimization variables, high coupling of multiple performance indices, and high velocity make the research difficult. Therefore, taking the cheetah-inspired quadruped robot as the research object, the method of changing the running direction of the robot was proposed to make the robot turn quickly in the process of high-speed movement and have good dynamic stability. (1) On the basis of establishing the dynamic model of the cheetah-inspired quadruped robot running, a two-level dynamic stability index system was proposed, including a minimum index system and a range index system, which cover almost all of the indices that affect the dynamic stability of the robot. (2) The optimization objective function based on the dynamic stability index system and optimization variables are determined. Then, the optimal values were obtained based on the improved bee colony algorithm. By controlling the leg posture parameters and the corresponding driving torques, the robot can change the motion direction during high-speed movement. (3) According to the method proposed in this paper, two examples were given: The robot turned 28.08° to the left and −28.08° to the right during forward running when viewed from the top view. The calculation results showed that the total inertia moment of the robot was in a small reasonable range, and the angular velocity of the robot at the moment of leaving the ground was approximately zero, which proved that the robot had good dynamic stability. The simulation results show that there is no obvious change in the posture of the trunk of the robot during the flight phase, and the robot can land stably, which also proved the correctness of the method. The method proposed in this paper can provide a theoretical basis for the realization of high-speed movement of the robot in 3D space and had good applicability.

## Figures and Tables

**Table 1:** Structural parameters of the cheetah-inspired quadruped robot.

Size of Thigh/[*r*, *h*]/m	Size of Calf/[*r*, *h*]/m	Size of Trunk/m	Mass of Thigh/Kg	Mass of Calf/Kg	Mass of Trunk/Kg	(*a*_2_, *b*_2_)/m
0.02/0.24	0.02/0.28	0.18 × 0.2 × 0.6	0.2	0.3	1.8	(0.12, 0.26)

**Table 2:** Known values during optimization for example 1.

*v*_1_/(m/s)	*v*_2_/(m/s)	(*x*_1_, *y*_1_, *z*_1_)/m	(*x*_2_, *y*_2_, *z*_2_)/m	Φ_0_/°	Ψ_0_/°	*Z_D_*/*Z_E_*/(Nm)
(0, 0, 0.22)	(1.73, 0.87, 3.26)	(0.13, 0.35, −0.2)	(0, 0.3, 0)	(25, 10, 0)	60	5.0/10

**Table 3:** Initial parameter ranges and optimization results for example 1.

Initial ranges	***h*_11_/m**		***h*_21_/m**		***d_k_*_1_/m**		***φ_k_*_1_/°**		***h*_12_/m**		***h*_22_/m**		***d_k_*_2_/m**	
	[0, 0.2]		[0, 0.3]		[−0.1, 0.1]		[0, 180]		[0, 0.6]		[0, 0.6]		[−0.1, 0.4]	
	***φ_k_*_2_/°**	***t*_1_/s**		***t*_2_/s**		***a*_12_**		***a*_22_**		***d*_12_/°**		***d*_22_/°**		***d*_32_/°**
	[0, 180]	[0.5, 1]		[0.1, 0.5]		[10, 61]		[0, 15]		[−15, 0]		[10, 45]		[−30, 30]
Optimization results	***h*_11_/m**		***h*_21_/m**		***d_k_*_1_/m**		***φ_k_*_1_/°**		***h*_12_/m**		***h*_22_/m**		***d_k_*_2_/m**	
	0.17		0.04		0.08		74.12		0.10		0.33		−0.02	
	***φ_k_*_2_/°**	***t*_1_/s**		***t*_2_/s**		***a*_12_**		***a*_22_**		***d*_12_/°**		***d*_22_/°**		***d*_32_/°**
	53.29	0.8		0.3		17		7.55		−13		32.69		20

**Table 4:** Known values during optimization for example 2.

*v*_1_/(m/s)	*v*_2_/(m/s)	(*x*_1_, *y*_1_, *z*_1_)/m	(*x*_2_, *y*_2_, *z*_2_)/m	Φ_0_/°	Ψ_0_/°	*Z_D_*/*Z_E_*/(Nm)
(0, 0, 0.25)	(−1.60, 0.80, 3.00)	(0.13, 0.35, −0.2)	(0, 0.3, 0)	(25, −10, 0)	60	5.0/10

**Table 5:** Initial parameter ranges and optimization results for example 2.

Initial ranges	***h*_11_/m**		***h*_21_/m**		***d_k_*_1_/m**		***φ_k_*_1_/°**		***h*_12_/m**		***h*_22_/m**		***d_k_*_2_/m**	
	[0, 0.3]		[0, 0.2]		[−0.1, 0.1]		[0, 180]		[0, 0.6]		[0, 0.6]		[−0.1, 0.4]	
	***φ_k_*_2_/°**	***t*_1_/s**		***t*_2_/s**		***a*_12_**		***a*_22_**		***d*_12_/°**		***d*_22_/°**		***d*_32_/°**
	[0, 180]	[0, 1]		[0, 0.5]		[10, 61]		[0, 15]		[−15, 0]		[−45, −10]		[−30, 30]
Optimization results	***h*_11_/m**		***h*_21_/m**		***d_k_*_1_/m**		***φ_k_*_1_/°**		***h*_12_/m**		***h*_22_/m**		***d_k_*_2_/m**	
	0.02		0.25		−0.13		0.05		0.03		0.15		0.001	
	***φ_k_*_2_/°**	***t*_1_**		***t*_2_**		***a*_12_**		***a*_22_**		***d*_12_/°**		***d*_22_/°**		***d*_32_/°**
	44.67	0.8		0.22		26.86		10		−15		−12		12.88

## Data Availability

The original data contributions presented in the paper are included in the article. Further inquiries can be directed to the corresponding authors.

## Supplementary Materials

The following supporting information can be downloaded at: https://www.mdpi.com/article/10.3390/s22249601/s1, Video S1: Simulation results of rapid steering in the running of the quadruped robot.
